# Diagnosis and Treatment of Drug-Resistant Epilepsy: Present and Future Perspectives

**DOI:** 10.3390/brainsci10110779

**Published:** 2020-10-26

**Authors:** Giovanni Assenza

**Affiliations:** Unit of Neurology, Neurophysiology, Neurobiology, Department of Medicine, University Campus Bio-Medico of Rome, via Álvaro del Portillo, 21, 00128 Rome, Italy; g.assenza@unicampus.it; Tel.: +39-06225411220; Fax: +39-06225411955

The introduction of the operative definition of drug-resistant epilepsy (DRE) was a turning point for clinicians and people with epilepsy (PwE). It is based on the indisputable phenomenon that resistance to antiseizure medications (ASM) is not dependent on the type of drugs when they are appropriately administrated. Indeed, after the failure of two different drugs, drug-resistance is announced [1]. The innovation was to provide clinicians and PwE with a practical schedule to quickly move to the next therapeutic level, i.e., surgery evaluation or neuromodulation, while continuing to try the hundreds of possible ASMs combinations to ameliorate the impact of seizures on PwE’s quality of life [2]. Its clinical relevance relies on the evidence that surgical treatment success is strictly dependent on the epilepsy disease duration, i.e., the earlier the surgery, the better the outcome [3].

Awfully, despite recent discoveries in precision medicine and innovative pharmacodynamic targets for medications, the portion of PwE facing drug-resistance did not change. One out of three PwE continues to suffer from seizures despite medications [4]. Several factors are engaged in establishing drug-resistance, from molecular changes in drug targets to inflammation [5], auto-immunity [6], structural abnormalities and genetic background. DRE realizes, through a complex combination of all of them, and the more concurrent factors we have, the more likely DRE is [7]. Focal epilepsy related to a structural lesion, high frequency of tonic-clonic seizures, and failure in seizure control after a first ASM are reliable and early factors predicting DRE. However, we did not dispose of an instrumental examination able to reliably predict DRE to date [8]. A new and feasible chance will be provided by big data and artificial intelligence analyses on clinical and EEG data [9,10], which could estimate the risk of DRE before starting the first ASM to offer the best therapy to each patient earlier, i.e., medication, surgery or neuromodulation.

Regarding therapeutic perspectives for DRE, the most rapidly developing and promising research field in epilepsy is represented by genetic. Its advances offer new hints at the pathogenesis of epilepsies, as recently demonstrated by the revision of the classification of epilepsies, promoted by the accumulating evidence that most of the generalized epilepsies subtend a genetic background [11]. Even if a huge number of genes are involved in the pathogenesis of epilepsy, the efforts of clinicians in categorizing distinct clinical profiles corresponding to single-gene mutations could, in future, open new opportunities for gene replacement therapies and for therapeutic choices driven by individualized genetic profiling.

Nowadays, after the ascertainment of the DRE, the treatment algorithm for PwE could offer only two choices, surgery or neuromodulation [2]. Surgical evaluation is the first step for focal onset epilepsies. Recently, Anyanwu and Motamedy [12] reviewed the clinical management of DRE, offering an exhaustive view of the comprehensive approach needed for a surgical plan. Once again, recent technological innovations provided a valuable contribution through the analysis of non-invasive EEG. Actually, the availability of high-density scalp caps increased the EEG topographical resolution power in localizing the intracranial source of epileptogenic zone. In recent years, electrical source imaging offered a non-redundant clinical support, reducing the need for special radiological techniques (such as PET and SPECT), and better guiding intracranial studies [13].

Regarding surgical approaches, it is worth mentioning the contribution of the magnetic resonance-guided laser interstitial thermal therapy. This is a new technology that provides a clinically efficacious and minimally invasive alternative to conventional microsurgical resections, with unquestionable advantages in terms of hospitalization duration and post-operative time recovery for PwE. Comparative clinical outcome data with respect to traditional surgery are progressively increasing and encouraging [14].

When surgery fails or is not feasible, neuromodulation is the only palliative choice for DRE. Vagal nerve stimulation, deep brain stimulation and responsive nerve stimulations have similar results in term of efficacy and possess different indications according to the type of epilepsy, offering different opportunities for DRE [2]. There is a huge scientific effort in studying the technological advancements of neuromodulation. The development of reliable closed-loop tools of stimulation is desirable for all these techniques to optimize efficacy, battery duration and adverse effects. To date, vagal nerve stimulation is furnished with an indirect electrocardiographic-driven closed-loop, which is suitable only for a portion of PwE, while responsive nerve stimulation has a reliable cortical closed-loop, and deep brain stimulation does not have a closed-loop system at all (even it is under development). 

In this light, important advancements could be provided by future developments of wearable devices, which could hopefully be implemented with neuromodulation techniques for closed-loop stimulation. Unfortunately, commercially available devices currently have fair accuracy in detecting convulsive seizures, but they cannot precisely identify focal non-motor seizures with or without awareness impairment [15]. However, wearable devices already represent accurate tools to precisely monitor vital signs (as electrocardiogram and blood oxygen saturation) in a continuous and non-invasive way, and thus they own a certain future clinical application in detecting severe seizures with cardio-pulmonary involvement and in preventing the Sudden Unexpected Death in Epilepsy (SUDEP).

Several non-invasive neuromodulation techniques are under evaluation with preliminary and promising data, such as transcranial direct stimulation [16,17], transcranial magnetic stimulation [18] and transcutaneous vagal nerve stimulation [19,20]. The published data are far from demonstrating their clinical efficacy on DRE, but the advantages for PwE would be so important that it is worth spending further efforts on their optimizations.

In conclusion, the diagnosis and treatment of DRE are at a turning point, coming from the absolute previous certainty of ASMs’ limitations, and are ready to be launched in a technology-driven future, that we hope could achieve surprising results for our PwE.
